# Enzymatic synthesis of nucleobase-modified UDP-sugars: scope and limitations

**DOI:** 10.1016/j.carres.2014.12.005

**Published:** 2015-03-02

**Authors:** Ben A. Wagstaff, Martin Rejzek, Thomas Pesnot, Lauren M. Tedaldi, Lorenzo Caputi, Ellis C. O’Neill, Stefano Benini, Gerd K. Wagner, Robert A. Field

**Affiliations:** a Department of Biological Chemistry, John Innes Centre, Norwich Research Park, Norwich NR4 7UH, UK; b School of Pharmacy, University of East Anglia, Norwich NR4 7TJ, UK; c King’s College London, Faculty of Natural & Mathematical Sciences, Department of Chemistry, Britannia House, 7 Trinity Street, London SE1 1DB, UK; d Bioorganic Chemistry and Bio-Crystallography laboratory, Faculty of Science and Technology, Free University of Bolzano, Piazza Università 5, 39100 Bolzano, Italy

**Keywords:** Sugar nucleotide, Modified nucleobase, Enzymatic synthesis, Pyrophosphatase, Epimerase

## Abstract

Glucose-1-phosphate uridylyltransferase in conjunction with UDP-glucose pyrophosphorylase was found to catalyse the conversion of a range of 5-substituted UTP derivatives into the corresponding UDP-galactose derivatives in poor yield. Notably the 5-iodo derivative was not converted to UDP-sugar. In contrast, UDP-glucose pyrophosphorylase in conjunction with inorganic pyrophosphatase was particularly effective at converting 5-substituted UTP derivatives, including the iodo compound, into a range of *gluco*-configured 5-substituted UDP-sugar derivatives in good yields. Attempts to effect 4″-epimerization of these 5-substituted UDP-glucose with UDP-glucose 4″-epimerase from yeast were unsuccessful, while use of the corresponding enzyme from *Erwinia amylovora* resulted in efficient epimerization of only 5-iodo-UDP-Glc, but not the corresponding 5-aryl derivatives, to give 5-iodo-UDP-Gal. Given the established potential for Pd-mediated cross-coupling of 5-iodo-UDP-sugars, this provides convenient access to the *galacto*-configured 5-substituted-UDP-sugars from *gluco*-configured substrates and 5-iodo-UTP.

## Highlights

• Enzymatic conversion of 5-aryl-substituted UTP to UDP-galactose derivatives.
• UDP-glucose pyrophosphorylase was particularly effective.
• Epimerization of 5-substituted UDP-glucoses with *Erwinia* UDP-glucose 4″-epimerase.

## Graphical abstract

**Figure:**
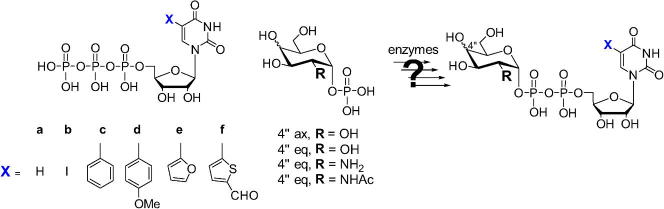

## 1 Introduction

**Figure 1:**
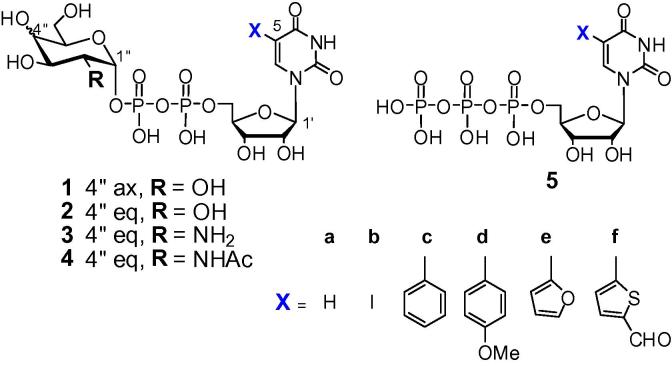
Target nucleobase-modified UDP-sugar derivatives (**1**)–(**4**) and UTP precursors (**5**).

Glycosyltransferases (GTs) are a large class of carbohydrate active enzymes that are involved in numerous important biological processes, with impact in cellular adhesion, carcinogenesis and neurobiology, amongst many others.^1–3^ As such, GTs have enormous potential as targets for drug discovery. For the full realization of this potential, both chemical inhibitors, and operationally simple and generally applicable GT bioassays, especially for high-throughput inhibitor screening, are indispensable tools.^4^ Many GTs use UDP-sugars as their donor substrates, and non-natural derivatives of these sugar-nucleotides are therefore of considerable interest as GT inhibitor candidates and assay tools.^5^ Wagner et al. have recently described 5-substituted UDP-sugars (Fig. 1) as a new class of GT inhibitors with a unique mode of action.^6–9^ Depending on the nature of the 5-substituent, these 5-substituted UDP-sugars also exhibit useful fluorescent properties,^10–12^ and we have recently reported a series of novel auto-fluorescent derivatives of UDP-sugars with a fluorogenic substituent at position 5 of the uracil base (Fig. 1).^10,11^ In a proof of concept study, Wagner et al. demonstrated that fluorescence emission by 5-formylthienyl-UDP-α-d-galactose (**1f**) is quenched upon specific binding to several retaining galactosyltransferases (GalTs), and that this effect can be used as a read-out in ligand-displacement experiments.^11^ To date, such 5-substituted UDP-sugar probes had to be prepared using chemical synthesis (reviewed in Ref. 5). For instance, Wagner et al. showed that it is possible to directly transform 5-iodo-UDP-α-d-Gal (**1b**) into 5-formylthienyl-UDP-α-d-Gal (**1f**) using Suzuki coupling under aqueous conditions.^6^ The aim of the current work was to explore alternative methods for the preparation of 5-substituted UDP-sugars (**1**–**4**) using chemo-enzymatic approaches (reviewed in Ref. 13) starting from 5-substituted UTP derivatives **5b**–**f**.^12^

## 2 Results and discussion

**Scheme 1:**
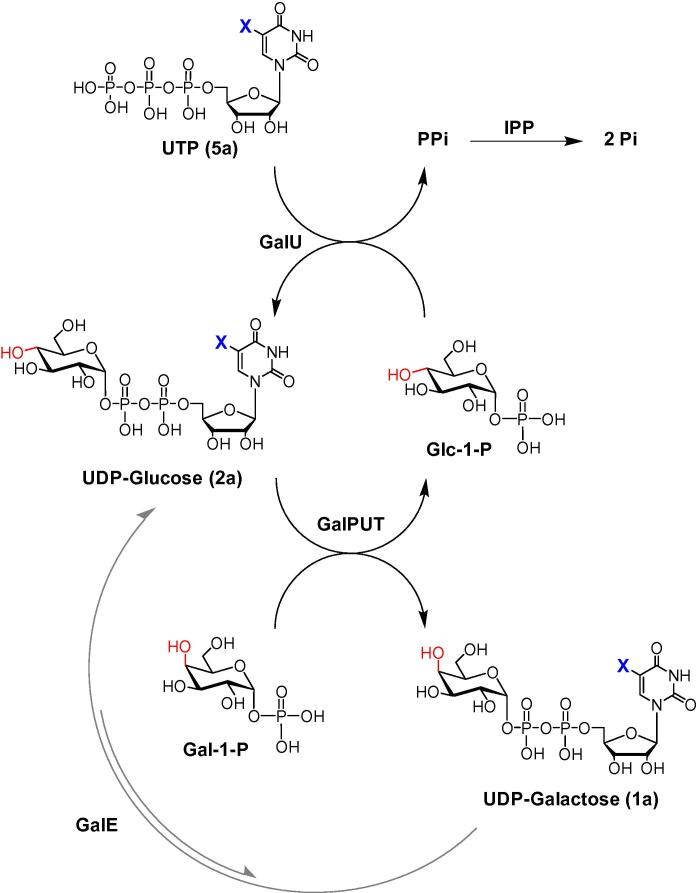
Strategies for enzymatic preparation of based-modified UDP-Glucose and UDP-Galactose. X is as outlined in Figure 1; GalPUT = galactose-1-phosphate uridylyltransferase; GalU = UDP-glucose pyrophosphorylase; GalE = UDP-Galactose 4″-epimerase; IPP = inorganic pyrophosphatase. Arrows in black indicate a one pot reaction; arrows in grey indicate a separate reaction.

Access to *gluco*- and *galacto*-configured UDP-sugars lies at the heart of this study. In brief, enzymatic synthesis approaches to such compounds may employ a number of different enzymes, either affording the required sugar nucleotide via pyrophosphate bond formation [action of uridylyltransferase (GalPUT) or pyrophosphorylase (GalU)], or by epimerization of the C-4″ stereochemistry of the pre-formed sugar nucleotide [action of epimerase (GalE)] (Scheme 1).^13^ We have employed both the former and latter approaches in syntheses of natural^14^ and non-natural^14–17^ sugar nucleotides.

### 2.1 Enzymatic synthesis of 5-substituted UDP-Gal derivatives using a GalU-GalPUT protocol

#### 2.1.1 One-pot GalU-GalPUT reactions

In an attempt to generate 5-substituted UDP-Gal derivatives **1b**–**f**, a multienzyme approach was assessed (Scheme 1).^14,15^ This protocol employs UTP (**5a**) and glucose-1-phosphate with UDP-glucose pyrophosphorylase (GalU, EC 2.7.7.9) to generate UDP-Glc (**2a**) in situ. Galactose-1-phosphate uridylyltransferase (GalPUT, EC 2.7.7.12) then catalyses the reaction of UDP-Glc (**2a**) and α-d-galactose-1-phosphate, giving the corresponding UDP-Gal (**1a**). UDP-Glc (**2a**) is only produced in catalytic quantity (typically 0.5 mol % related to sugar-1-phosphate) as it is continuously recycled, via Glc-1-P, by the action of GalU (Scheme 1). In this reaction, inorganic pyrophosphate is released and inorganic pyrophosphatase (IPP) is employed to achieve its hydrolysis, driving the overall equilibrium of the multi-enzyme reaction towards the formation of the desired UDP-Gal (**1a**) sugar nucleotide.

In a control experiment, Gal-1-P was converted into UDP-Gal (**1a**) using an equimolar quantity of UTP (**5a**) and a catalytic amount of UDP-Glc (**2a**). The transformation reached a complete conversion (by SAX HPLC) within 1 h (data not shown). Next, the 5-substituted UTP derivatives **5b**–**f** were used in combination with Gal-1-P in an attempt to generate the corresponding 5-substituted UDP-Gal derivatives **1b**–**f** (Fig. 1; Scheme 1). In all cases, reaction with the 5-substituted UTPs was slower than with the parent compound. After 24 h incubation, formation of product was detected in the case of 5-(4-methoxyphenyl)-UDP-Gal (**1d**) (5%) and 5-(2-furyl)-UDP-Gal (**1e**) (23%) and the products were isolated and characterized. The formation of 5-(4-methoxyphenyl)-UDP-Glc (**2d**) and 5-(2-furyl)-UDP-Glc (**2e**) as intermediates in the reaction is implicit, but their presence in the reaction mixture was not detected. The 5-iodo-UTP (**5b**) and the 5-(5-formyl-2-thienyl)-UTP (**5f**) derivatives were not converted into the corresponding sugar nucleotides at all; in the case of the 5-phenyl-UTP (**5c**), the conversion was less than 5% and the product **1c** was not isolable. In order to assess which of the enzymes, GalU or GalPUT, is failing to use these latter base-modified compounds as substrates, a series of reverse reactions and inhibition experiments was performed.

#### 2.1.2 The GalPUT reaction in reverse

**Figure 2:**
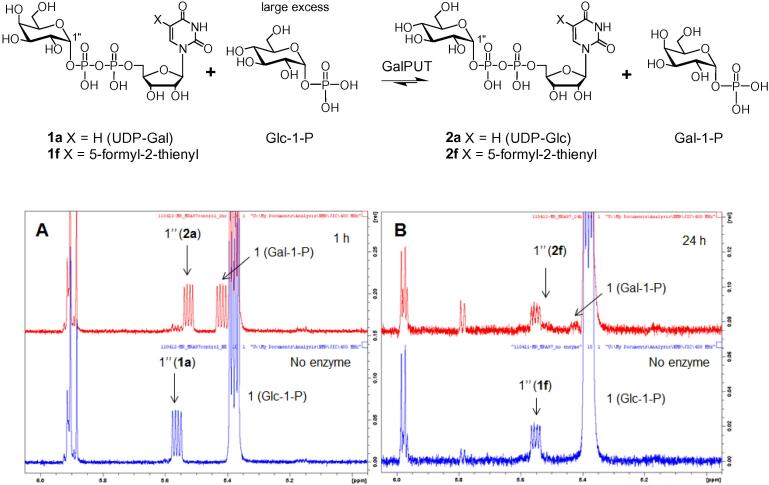
Reverse action of GalPUT. (A) Incubation of UDP-Gal (**1a**) and Glc-1-P. (B): Incubation of 5-(5-formyl-2-thienyl)-UDP-Gal (**1f**) and Glc-1-P.

In the presence of excess Glc-1-P, GalPUT can be used to run the reverse conversion, UDP-Gal (**1a**) into UDP-Glc (**2a**). As shown in Figure 2A, after 1 h the conversion of substrate into product is nearly complete, as judged by ^1^H NMR analysis of the diagnostic anomeric signals (dd) of the sugar phosphates. When a synthetic sample of 5-(5-formyl-2-thienyl)-UDP-Gal (**1f**) was subjected to equivalent conditions, no conversion was observed after 1 h (data not shown). After extended incubation (24 h) only traces of 5-(5-formyl-2-thienyl)-UDP-Glc (**2f**) and Gal-1-P were detectable by ^1^H NMR (Fig. 2B). This result suggests that 5-(5-formyl-2-thienyl)-UDP-Gal (**1f**) either does not bind to the GalPUT active site or that it might bind in a non-productive way. If the latter were true, **1f** should act as a GalPUT inhibitor.

#### 2.1.3 5-(5-Formyl-2-thienyl)-UDP-Gal as a GalPUT inhibitor

When UDP-Gal (**1a**), Glc-1-P and **1f** were mixed in a molar ratio 1:10:3.5, the conversion to UDP-Glc (**2a**) after 30 min was the same as in the absence of **1f** (not shown), implying that **1f** does not compete with UDP-Gal (**1a**) to bind in the active site of GalPUT. This implies that the formylthienyl substitution of the uracil base prevents the corresponding sugar nucleotides from binding to GalPUT and explains the observed lack of conversion of **2f** into **1f** in the one-pot GalU-GalPUT protocol. However, the lack of conversion might also be due to a lack of tolerance of GalU for 5-substitution of the uracil ring of UTP.

#### 2.1.4 Competing 5-substituted-UTP and unsubstituted UTP as GalU substrates

**Figure 3:**
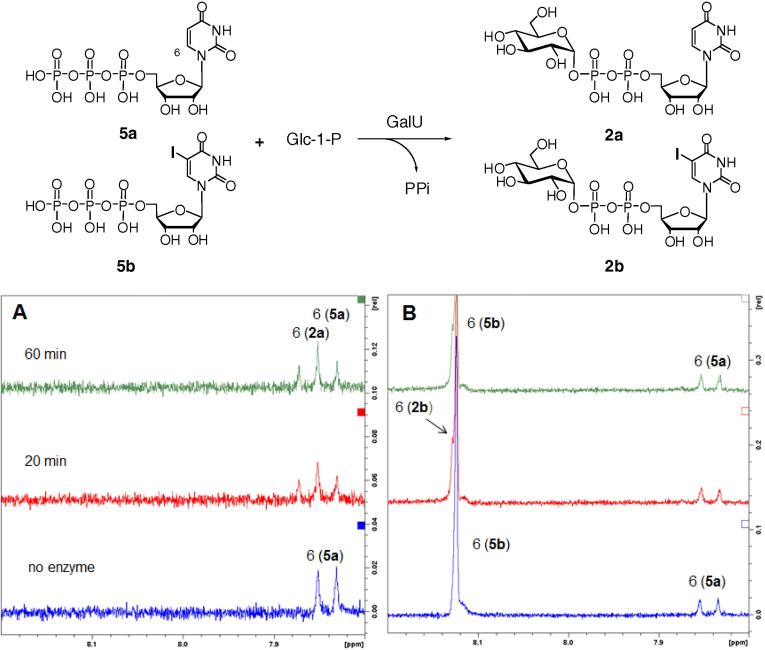
UTP (**5a**) and 5I-UTP (**5b**) competition in GalU mediated conversion of Glc-1-P into the corresponding sugar nucleotides. (A) control with UTP (**5a**) only. (B) UTP (**5a**) and large excess 5I-UTP (**5b**).

A series of experiments were conducted to assess the flexibility of GalU towards 5-substitution of its UTP substrate. A control experiment [GalU, Glc-1-P, UTP (**5a**)] showed the rapid conversion of UTP (**5a**) into UDP-Glc (**2a**), as indicated by the diagnostic uracil H6 signals in ^1^H NMR spectra (Fig. 3A). In a competition experiment employing Glc-1-P, UTP (**5a**) and 5-iodo-UTP (**5b**) in molar ratio 1:1:5, UTP (**5a**) remained almost completely intact (Fig. 3B). Instead, 5-iodo-UTP (**5b**) was rapidly converted into 5-iodo-UDP-Glc (**2b**), as shown by a new H6 signal (Fig. 3B) and confirmed by LC–MS: a molecular ion for 5-iodo-UDP-Glc (**2b**) ([M−H]^−^ *m*/*z* 691) was detected, but one for UDP-Glc (**2a**) ([M−H]^−^ *m*/*z* 565) was absent. These data suggest that, if used in excess, 5-iodo-UTP (**5b**) can out-compete UTP (**5a**), the natural substrate of GalU, indicating some degree of relaxed GalU substrate specificity. The fact that no formation of 5-iodo-UDP-Gal (**1b**) was observed in the multi-enzyme transformation (Scheme 1) suggests that although a small quantity of 5-iodo-UDP-Glc (**2b**) may have been formed in that reaction, it could not be further processed by GalPUT.

### 2.2 Enzymatic synthesis of 5-substituted UDP-Glc **2b**–**f** using GalU

#### 2.2.1 GalU reactions with substituted UTPs

**Figure 4:**
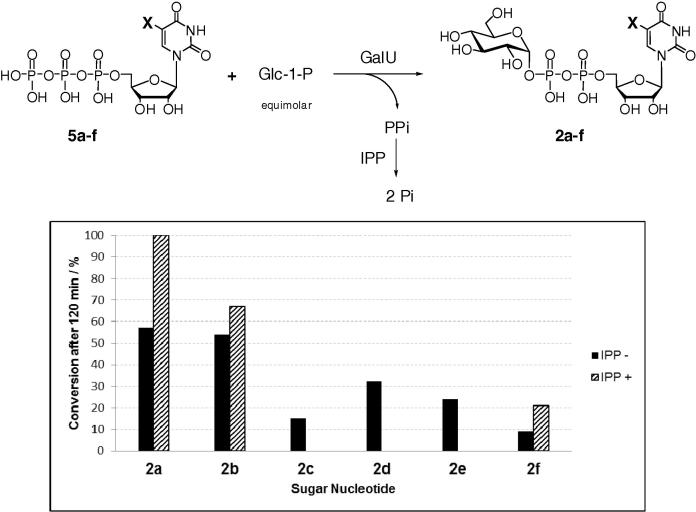
GalU-mediated formation of 5-substituted UDP-Glc derivatives in the absence of IPP (time point 120 min). X indicates: **a** = H, **b** = I, **c** = Ph, **d** = 4-MeO-Ph, **e** = 2-furyl, **f** = 5-formyl-2-thienyl.

The results presented above indicate that GalU possesses a degree of substrate flexibility regarding 5-substitution of UTP, potentially offering easy access to 5-substituted UDP-Glc (**2b**–**f**) derivatives. This was indeed the case when 5-substituted UTP derivatives **5b**–**f** and an equimolar amount of Glc-1-P were subjected to GalU (Fig. 4). Conversions to the corresponding sugar nucleotides **2b**–**f** ranged from 9% to 54% after 120 min. Unsurprisingly, the lowest conversion was detected for the bulky 5-(5-formyl-2-thienyl)-derivative **2f**. A control reaction of UTP (**5a**) with Glc-1-P under the same conditions gave 57% conversion to UDP-Glc (**2a**). When inorganic pyrophosphatase (IPP) was added to reactions, the conversions could be further improved (Fig. 4).

**Figure 5:**
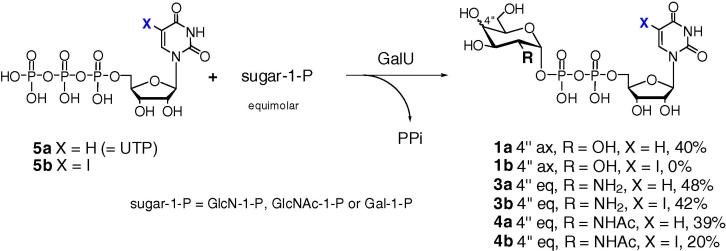
GalU mediated transformations of UTP (**5a**) and 5I-UTP (**5b**) with three different sugar-1-phosphates (Gal-1-P, GlcN-1-P or GlcNAc-1-P) to the corresponding sugar nucleotides **1**, **3** and **4** at time point 120 min.

Under these conditions, the conversion of **5f** into the 5-(5-formyl-2-thienyl)-derivative **2f** was a tolerable 21%. GalU also showed remarkable substrate flexibility towards the configuration of sugar-1-phosphates^18^—a feature that it has in common with other pyrophosphatases, such as RmlA.^19,20^ When UTP (**5a**) was employed as a co-substrate, GalU proved capable of accepting α-d-glucosamine-1-phosphate (GlcN-1-P) and *N*-acetyl-α-d-glucosamine-1-phosphate (GlcNAc-1-P), as well as α-d-galactose-1-phosphate (Gal-1-P) (Fig. 5). Conversions to the corresponding UDP-sugars **1a**, **3a** and **4a**, respectively, reached 39–48% after 120 min (Fig. 5). With 5-iodo-UTP (**5b**) the GalU-mediated conversions were lower in the case of GlcN-1-P (42%) and GlcNAc-1-P (20%); disconcertingly, no conversion at all was detected in the case of Gal-1-P (Fig. 5).

#### 2.2.2 The mutual incompatibility of 5-iodo-UTP and Gal-1-P as co-substrates for GalU

**Figure 6:**
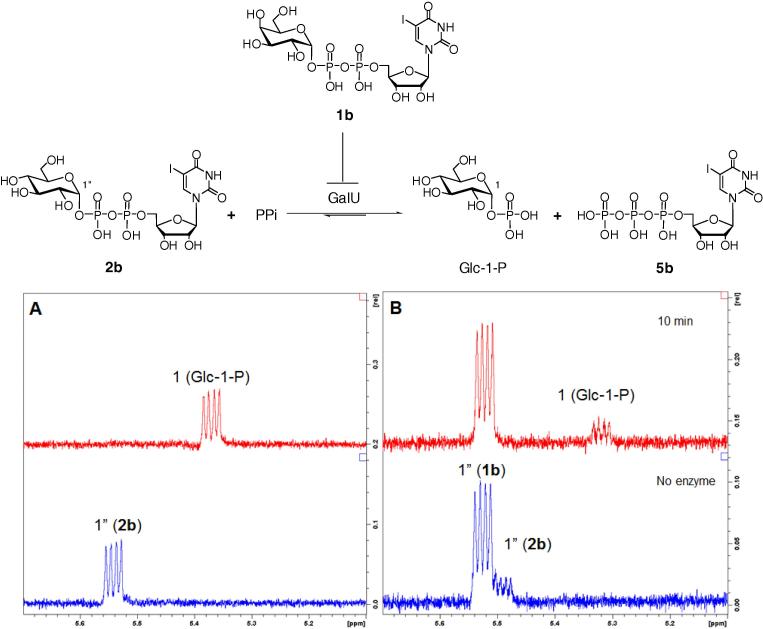
Reverse action of GalU in the presence of excess 10 mM PPi. (A) Conversion of 5-iodo-UDP-Glc (**2b**) into Glc-1-P. (B) The same as A, but in the presence of excess (5 equiv) 5-iodo-UDP-Gal (**1b**).

The lack of GalU-mediated conversion of Gal-1-P with 5-iodo-UTP was somewhat unexpected and warranted further analysis. First, it was shown that in the presence of a high concentration of inorganic pyrophosphate (PPi), GalU can perform the reverse conversion from 5-iodo-UDP-Glc (**2b**) to Glc-1-P and 5-iodo-UTP (**5b**). The conversion was complete, as judged by ^1^H NMR [anomeric proton resonances (dd) were used as diagnostic peaks], within 10 min with 10 mM PPi (Fig. 6). When 5-iodo-UDP-Gal (**1b**) was subjected to analogous conditions no conversion was observed even after incubation for 60 min (data not shown). To see whether the lack of conversion of the *galacto*-configured substrates was down to lack of binding or to non-productive binding of the substrates, inhibition experiments employing 5 equiv of 5-iodo-UDP-Gal (**1b**), 1 equiv of 5-iodo-UDP-Glc (**2b**) and excess PPi were conducted. By ^1^H NMR, 5-iodo-UDP-Glc (**2b**) was fully converted into Glc-1-P and 5-iodo-UTP (**5b**) within 10 min as in the no inhibitor control reaction (Fig. 6). No conversion 5-iodo-UDP-Gal (**1b**) was detected, which suggests that 5-iodo-UDP-Gal (**1b**) does not bind to the active site of GalU. A competition experiment was designed to show whether a large excess of Gal-1-P (5 equiv) can outcompete the natural acceptor Glc-1-P (1 equiv) in a GalU mediated conversion of 5-iodo-UTP (**5b**) (1 equiv) into 5-iodo-UDP-sugar. ^1^H NMR spectra showed that only 5-iodo-UDP-Glc (**2b**) was formed and no trace of 5-iodo-UDP-Gal (**1b**) was detected, even after 120 min (not shown).

From the above data, it is evident that GalU is not able to simultaneously bind both Gal-1-P and 5-iodo-UTP (**5b**), although both in their own right are productive substrates in the presence of alternative co-substrates. It may be that a conformational change is required in order to enable co-substrates to bind to GalU in a productive manner, but this is either too slow, or it does not happen at all, when Gal-1-P and 5-iodo-UTP are employed. Further structural analyses are required in order to address this point.

### 2.3 Enzymatic epimerization of 5-iodo-UDP-Glc (**2b**) to give the corresponding 5-iodo-UDP-Gal (**1b**) using GalE

As noted above, GalU successfully produces a range of base-modified *gluco*-configured UDP-sugars but fails to produce the corresponding *galacto*-configured compound. The one-pot, GalU-GalPUT protocol showed some flexibility, producing *galacto*-configured analogues **1d** and **1e** in low yield, but **1b**, **1c** and **1f** were not accessible by this route. An alternative approach to the *galacto*-configured series is an epimerization of 4″-OH in the base-modified UDP-Glc derivatives. Uridine-5′-diphosphogalactose 4″-epimerase (GalE, E.C. 5.1.3.2) is an enzyme known to catalyse the conversion of UDP-Gal (**1a**) into UDP-Glc (**2a**), with the equilibrium favouring the latter over the former (ca 1:4).^21^ Previous work suggested that 5-formylthienyl-UDP-Gal (**1f**) is not a substrate for *Streptococcus thermophilus* GalE.^22^ Therefore GalE from two further organisms was assessed: galactose-adapted yeast (*Sc*GalE)^23^ and *Erwinia amylovora* (*Ea*GalE).^24^

**Figure 7:**
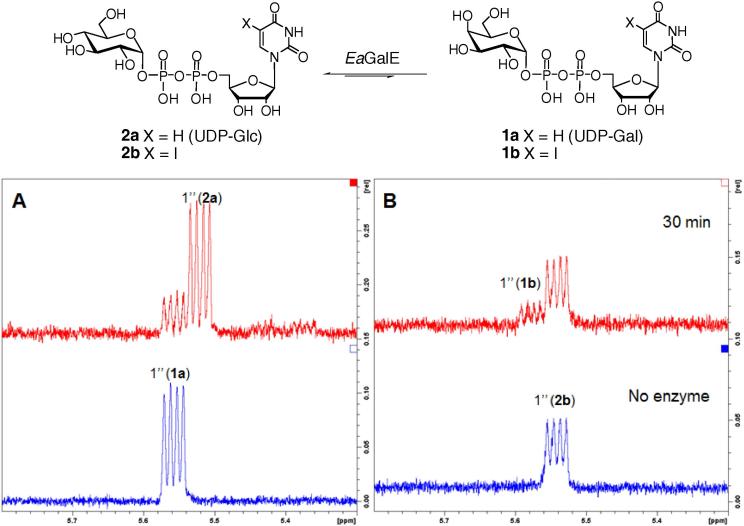
*Ea*GalE mediated epimerization. (A) UDP-Gal (**1a**) into UDP-Glc (**2a**) (30 min). (B) 5-Iodo-UDP-Glc (**2b**) into 5-iodo-UDP-Gal (**1b**) (30 min).

As control experiments, the conversion of UDP-Gal (**1a**) into UDP-Glc (**2a**) was achieved using both *Sc*GalE and *Ea*GalE and the progress of the epimerization was followed by ^1^H NMR. Under the condition employed, the equilibrium reaction mixtures were reached within 10 min and the ratio between *galacto*-/*gluco*-configured products were approximately 1:4, as expected (Fig. 7). Treatment of 5-formylthienyl-UDP-Gal (**1f**) with *Sc*GalE and *Ea*GalE did not show any 4″-OH epimerization by ^1^H NMR, even after prolonged incubation (120 min). Similarly, when 5-iodo-UDP-Gal (**1b**) was used as a substrate, *Sc*GalE failed to effect conversion, even after extended incubation (120 min).

However, in contrast, *Ea*GalE showed rapid epimerization of 5-iodo-UDP-Gal (**1b**) into 5-iodo-UDP-Glc (**2b**) and the transformation reached equilibrium after about 30 min giving mixed *galacto*-/*gluco*-configured products in the ratio 3:7. The reverse conversion of **2b** into **1b** using *Ea*GalE was also shown to achieve a ca 7.5:2.5 equilibrium mixture of *gluco*-/*galacto*-configured sugar nucleotides after 30 min (Fig. 7).

## 3 Conclusions

5-Substituted *gluco*- and *galacto*-configured UDP-sugars are versatile tools for glycoscience research. To date, access to such compounds has relied on chemical synthesis approaches. Here we have investigated enzymatic synthesis routes to such compounds, relying either on pyrophosphate bond formation [action of uridylyltransferase (GalPUT) or pyrophosphorylase (GalU)] or epimerization of the C-4″ stereochemistry of the pre-formed sugar nucleotide [action of epimerase (GalE)]. These studies demonstrate that the one-pot combination of glucose-1-phosphate uridylyltransferase (GalPUT) and UDP-glucose pyrophosphorylase (GalU) is able to catalyse the conversion of 5-substituted UTP derivatives into the corresponding 5-substituted UDP-galactose derivatives in a number of instances, albeit in poor yield (<5–23% isolated yield). It appears that the specificity of GalPUT is a limiting factor in the utility of this reaction. In contrast, GalU in conjunction with inorganic pyrophosphatase was able to convert 5-substituted UTP derivatives plus a range of *gluco*-configured sugar-1-phosphates into the corresponding sugar nucleotides in practical yields (20–98%). Subsequent attempts to convert these *gluco*-configured compounds to the corresponding *galacto*-isomers proved problematic, with UDP-glucose 4″-epimerase (GalE) from both yeast and Erwinia proving ineffective for bulky 5-aryl derivatives. However, in contrast to the yeast enzyme, the Erwinia GalE proved effective with 5-iodo-UDP-glucose, readily converting it to 5-iodo-UDP-galactose. Given the established potential for Pd-mediated cross-coupling of 5-iodo-UDP-sugars, the enzymatic procedures elaborated in this study provide useful additions to the repertoire of transformation available for the production of novel sugar nucleotides.

## 4 Experimental

### 4.1 General methods

#### 4.1.1 Chemicals

All chemicals and reagents were obtained commercially and used as received unless stated otherwise. The identity of products from our control experiments (**1a**, **2a** and **4a**) was confirmed by comparison of ^1^H NMR spectra and/or HPLC retention times of authentic samples. 5-Substituted UTP derivatives **5b**–**f**^12^ and 5-iodo-UDP-Gal (**1b**), 5-iodo-UDP-Glc (**2b**) and 5-(5-formyl-2-thienyl)-UDP-Gal (**1f**)^7^ were prepared by chemical synthesis following published procedures. The identity of the following known compounds were confirmed by comparison of analytical data with published literature: (**1b**),^7^ (**1d**),^7^ (**1e**),^7^ (**2b**),^10^ (**2c**),^10^ (**2d**),^10^ (**2e**),^10^ (**3a**),^25^ (**4b**).^6^

#### 4.1.2 Spectroscopy

^1^H NMR spectra were recorded in D_2_O on a Bruker Avance III spectrometer at 400 MHz and chemical shifts are reported with respect to residual HDO at *δ*_H_ 4.70 ppm. High resolution accurate mass spectra were obtained using a Synapt G2 Q-Tof mass spectrometer using negative electrospray ionization. Low resolution mass spectra were obtained using either a Synapt G2 Q-Tof or a DecaXPplus ion trap in ESI negative mode by automated direct injection.

#### 4.1.3 Enzymes

Galactose-1-phosphate uridylyltransferase (GalPUT, EC 2.7.7.12) from *Escherichia coli* was over-expressed and purified as described earlier.^14^ Glucose-1-phosphate uridylyltransferase (GalU) from *Escherichia coli* was over-expressed and purified as described earlier.^26^ Inorganic pyrophosphatase (IPP) from *Saccharomyces cerevisiae* was purchased from Sigma–Aldrich. Uridine-5′-diphosphogalactose 4″-epimerase (GalE) from galactose-adapted yeast (*Saccharomyces cerevisiae*, *Sc*GalE) was purchased from Sigma–Aldrich. Uridine-5′-diphosphogalactose 4″-epimerase (GalE) from *Erwinia amylovora* (*Ea*GalE) was cloned, overexpressed and purified as detailed below.

#### 4.1.4 Erwinia amylovora (EaGalE)

The GalE gene (ENA accession number FN666575.1) was amplified by PCR from genomic DNA isolated from *E. amylovora* strain Ea273 (ATCC 49946) using the following primers: GalE-F 5′-CGATCACCATGGCTATTTTAGTCACGGGGG and GalE-R 5′-CGATCACTCGAGTCAACTATAGCCTTGGGG. These primers included NcoI and XhoI restriction sites, respectively (underlined). The PCR product was purified from agarose gel using a QIAquick gel extraction kit (Qiagen, Germany) and treated for 3 h at 37 °C with NcoI and XhoI (NEB, USA) for double digestion. After purification using QIAquick PCR Purification Kit (Qiagen, Germany), the digested PCR product was ligated into pETM-30 vector.^27^ The construct was propagated in *Escherichia coli* NovaBlue cells (EMD4Biosciences, Germany), purified using a DNA miniprep kit (Sigma, USA) and sequenced by Microsynth AG (Switzerland) to test the correctness of the gene sequence. *E. coli* BL21 (DE3) chemically competent cells (EMD4Biosciences, Germany) were transformed with the pETM-30::GalE construct for expression of the recombinant GST-fusion protein. Cells containing the construct were grown overnight in 10 mL 2 × YT medium containing Kanamycin (30 μg mL^−1^) at 37 °C. The starter culture was used to seed 1 L of medium (1:100 dilution) and the culture was grown at 37 °C for 3 h (O.D. 0.8). The temperature was then decreased to 18 °C and the culture was left to equilibrate for 1 h before induction with 1 mM IPTG for 16 h. Cells were harvested by centrifugation at 4500*g* for 15 min at 4 °C, re-suspended in 50 mL ice cold PBS containing 0.2 mg mL^−1^ lysozyme and protease inhibitors, stirred for 30 min at room temperature and lysed by sonication (Soniprep, MSE, UK) on ice for 2 min using 2 s cycles (15.6 MHz). After centrifugation at 18000*g* for 20 min at 4 °C the supernatant was filtered and loaded onto a GSTrap HP 5 mL column (GE Healthcare, Sweden) equilibrated with PBS at a flow rate of 1.5 mL min^−1^. The column was then washed with PBS until the *A*_280_ reached the baseline and the enzyme was eluted with 10 mM reduced glutathione in 50 mM TRIS–HCl buffer at pH 8.0. The eluted protein was dialysed against 50 mM TRIS–HCl buffer at pH 8.0 containing 10% glycerol, concentrated to 0.1 mg mL^−1^ and stored at −20 °C. Protein purity was confirmed by SDS–PAGE.

### 4.2 Sugar nucleotide purification methods

#### 4.2.1 Purification method 1

Strong anion-exchange (SAX) HPLC on Poros HQ 50. An aqueous solution of a sample was applied on a Poros HQ 50 column (L/D 50/10 mm, CV = 3.9 mL). The column was first equilibrated with 5 CV of 5 mM ammonium bicarbonate buffer, followed by a linear gradient of ammonium bicarbonate from 5 mM to 250 mM in 15 CV, then hold for 5 CV, and finally back to 5 mM ammonium bicarbonate in 3 CV at a flow rate of 8 mL/min and detection with an on-line detector to monitor A_265_. After multiple injections, the column was washed with 3 CV of 1 M ammonium bicarbonate followed by 3 CV of MQ water.

#### 4.2.2 Purification method 2

Reverse phase (RP) C18 purification. The purification was performed on a Dionex Ultimate 3000 instrument equipped with UV/vis detector. A solution of a sample in water was applied on a Phenomenex Luna 5 μm C18(2) column (L/D 250/10 mm, CV = 19.6 mL) and eluted isocratically with 50 mM Et_3_NHOAc, pH 6.8 with 1.5% CH_3_CN in 8 CV at a flow rate of 5 mL/min and detection with on-line UV detector to monitor A_265_. Fractions containing the sugar nucleotide were pooled and freeze-dried.

### 4.3 Enzymatic transformations

#### 4.3.1 General procedure 1 (GalU-GalPUT-IPP)

UTP analogue (**5a**–**f**, 0.5 mg, 1 equiv), α-d-galactose-1-phosphate (1 equiv) and UDP-Glc (**2a**, 0.5 mol-%) were dissolved in buffer (500 μL, 50 mM HEPES pH 8.0, 5 mM KCl, 10 mM MgCl_2_). A small sample (50 μL) was separated for no enzyme control. Then enzymes were added to give final concentration of GalU (137 μg/mL), IPP (1.4 U/mL) and GalPut (329 μg/mL) in a final volume of 700 μL. The mixture was incubated at 37 °C with gentle shaking. At time points analytical samples were separated (50 μL) and MeOH was added (50 μL) to precipitate the enzymes. The sample was vortexed for 1 min, centrifuged (10,000 rpm for 5 min) and the supernatant was filtered through a disc filter (0.45 μm). The filtrate was analysed by SAX HPLC (10 μL injection, Purification method 1). After 24 h the reaction was quenched by addition of MeOH (the same volume as the sample volume) and processed as indicated for the analytical sample. Products were isolated using SAX HLC (Purification method 1). Pooled fractions containing the sugar nucleotide were freeze-dried. When necessary, Purification method 2 was also applied.

#### 4.3.2 General procedure 2 (GalU)

UTP analogue (**5a**–**f**, 0.5 mg, 1 equiv) and sugar-1-phosphate (1 equiv) were dissolved in deuterated buffer (660 μL, 50 mM HEPES pD 8.0, 5 mM KCl, 10 mM MgCl_2_) and ^1^H NMR was acquired (100 scans) of the no enzyme control. GalU (40 μL, final *c* = 0.14 mg/mL) was added to total 0.7 mL. Where indicated IPP (10 μL, final *c* = 1.4 U/mL) was added to this mixture. ^1^H NMR spectra were acquired at time points 10, 30, 60, 120 min to monitor reaction progress. The reaction was quenched by addition of an equal volume of methanol (0.7 mL), and the resulting solution was filtered through a 0.22 μm disc filter and products were purified using Purification method 1.

#### 4.3.3 General procedure 3 (GalE)

Appropriate sugar nucleotide (**1a**, **1b**, **1f**, or **2b**, 0.5 mg) was dissolved in deuterated buffer (660 μL, 50 mM HEPES pD 8.0, 5 mM KCl, 10 mM MgCl_2_) and ^1^H NMR of no enzyme control was acquired (100 scans). GalE was added (40 μL, final *c* = 36.6 μg/mL for *Ea*GalE, 140 μg/mL for *Sc*GalE) to give final volume of 0.7 mL and ^1^H NMR spectra were acquired at time points 10, 30 and 60 min.

##### 4.3.3.1 5-(5-Formyl-2-thienyl)-UDP-α-d-glucose (**2f**)

The title compound 2f was prepared from 5-(5-formyl-2-thienyl)-UTP (**5f**, 0.5 mg, 0.56 μmol) and Glc-1-P (0.17 mg, 0.56 μmol) as described in General procedure 2 and the product was isolated using Purification method 1 followed by Purification method 2 with the following modification: isocratic elution for 20 min at flow 5 mL/min with 50 mM Et_3_N.HOAc, pH 6.8 with 1.5% (93%, solvent A) and acetonitrile (7%, solvent B), UV detection at 350 and 265 nm. The title compound **2f** eluted at *R_f_* = 9.5 min and was obtained after freeze-drying as bistriethylammonium salt (∼0.01 mg, 1.7 %).The diagnostic peaks were extracted from a spectrum of the crude mixture purified by SAX only (Purification method 1) giving bisammonium salt of **2f**. ^1^H NMR (400 MHz, D_2_O): *δ* 9.69 (1H, s, CHO), 8.32 (1H, s, H-6), 7.92 (1H, d, ^3^*J*_Th3,Th4_ = 4.2 Hz, Th), 7.64 (1H, d, ^3^*J*_Th3,Th4_ = 4.3 Hz, Th), 6.01 (1H, d, ^3^*J*_1′,2′_ = 4.7 Hz, H-1′), 5.51 (1H, dd, ^3^*J*_1″,2″_ = 3.4 Hz, ^3^*J*_1″,Pβ_ = 7.3 Hz, H-1″). HRMS, ESI negative: *m*/*z* calcd for C_20_H_25_N_2_O_18_P_2_S^−^ [M−H]^−^: 675.0304, found: 675.0304.

##### 4.3.3.2 5-Iodo-UDP-α-d-glucosamine (**3b**)

The title compound **3b** was prepared from 5-iodo-UTP (**5b**, 0.5 mg, 0.56 μmol) and GlcN-1-P (0.15 mg, 0.56 μmol) as described in General procedure 2 and the product was isolated using Purification method 1 followed by Purification method 2. The title compound **3b** eluted at *R*_f_ = 12.9 min and was obtained after freeze-drying as a triethylammonium salt (**3b** × 0.5 Et_3_N, 0.1 mg, 20 %). ^1^H NMR (400 MHz, D_2_O): *δ* 8.13 (1H, s, H-6), 5.85 (1H, d, ^3^*J*_1′,2′_ = 3.7 Hz, H-1′), 5.66–5.60 (1H, m, H-1″), 4.31–4.27 (2H, m, H-2′, H-3′), 4.21–4.12 (3H, m, H-5a′, H-5b′, H-4′), 3.87–3.66 (5H, m, H-2″, H-3″, H-5″, H-6a″, H-6b″), 3.45–3.39 (1H, m, H-4″), 3.19 (3H, q, ^3^*J*_CH2,CH3_ = 6.8 Hz, (CH_3_C*H*_2_)_3_N), 1.17 (4.5H, t, ^3^*J*_CH2,CH3_ = 6.8 Hz, (C*H*_3_CH_2_)_3_N). HRMS, ESI negative: *m*/*z* calcd for C_15_H_23_IN_3_O_16_P_2_^−^ [M−H]^−^: 689.9604, found: 689.9596.
